# Stigma in response to mental disorders: a comparison of Australia and Japan

**DOI:** 10.1186/1471-244X-6-21

**Published:** 2006-05-23

**Authors:** Kathleen M Griffiths, Yoshibumi Nakane, Helen Christensen, Kumiko Yoshioka, Anthony F Jorm, Hideyuki Nakane

**Affiliations:** 1 Centre for Mental Health Research, The Australian National University, Canberra, ACT, 0200, Australia; 2 Department of Social Work, The Faculty of Human Sociology, Nagasaki International University, 2825-7 Huis Ten Bosch-cho, Sasebo-shi, Nagasaki, 859-3298, Japan; 3 ORYGEN Research Centre, Department of Psychiatry, University of Melbourne, Locked Bag 10, Parkville, Victoria, 3052, Australia; 4 Division of Neuropsychiatry, Department of Translational Medical Sciences, Nagasaki University Graduate School of Biomedical Sciences, 1-7-1 Sakamoto, Nagasaki, 852-8501, Japan

## Abstract

**Background:**

There are few national or cross-cultural studies of the stigma associated with mental disorders. Australia and Japan have different systems of psychiatric health care, and distinct differences in cultural values, but enjoy similar standards of living. This study seeks to compare the nature and extent of stigma among the public in the two countries.

**Methods:**

A household survey of the public was conducted in each country using similar methodologies. The Australian study comprised a national survey of 3998 adults aged over 18 years. The Japanese survey involved 2000 adults aged 20 to 69 from 25 regional sites distributed across the country. Interviewees reported their personal attitudes (personal stigma, social distance) and perceptions of the attitudes of others (perceived stigma, perceived discrimination) in the community with respect to four case vignettes. These vignettes described a person with: depression; depression with suicidal ideation; early schizophrenia; and chronic schizophrenia.

**Results:**

Personal stigma and social distance were typically greater among the Japanese than the Australian public whereas the reverse was true with respect to the perception of the attitudes and discriminatory behaviour of others. In both countries, personal stigma was significantly greater than perceived stigma. The public in both countries showed evidence of greater social distance, greater personal stigma and greater perceived stigma for schizophrenia (particularly in its chronic form) than for depression. There was little evidence of a difference in stigma for depression with and without suicide for either country. However, social distance was greater for chronic compared to early schizophrenia for the Australian public.

**Conclusion:**

Stigmatising attitudes were common in both countries, but negative attitudes were greater among the Japanese than the Australian public. The results suggest that there is a need to implement national public awareness interventions tailored to the needs of each country. The current results provide a baseline for future tracking of national stigma levels in each country.

## Background

Stigma has been identified by policy makers, professional and non-government organisations, researchers and consumers as a key issue in mental illness ([1-7]). Stigmatising attitudes may inhibit help seeking among individuals with a mental disorder [8,9], provide barriers to their successful reintegration into society [10], and increase their psychological distress [11].

Despite the importance of stigma, there have been few systematic national studies of the prevalence and nature of public attitudes to mental illness. One approach to better understanding stigma is to conduct comparative studies across countries that differ in the cultural contexts in which attitudes to mental illness form. A number of cross-cultural comparisons of attitudes to mental illness exist (eg, [12-18]), but with few exceptions (eg, [19]), these studies have involved relatively small, select samples derived from a local area or specific health or educational provider or student samples. Despite the markedly different cultural contexts involved, there is little empirical data contrasting attitudes to mental illness in Asian compared to Western countries.

Accordingly, in this paper we report a comparative study of the stigma associated with mental disorders in Japan and Australia. It is frequently assumed that stigma directed towards people with a mental disorder is more prevalent amongst the public in Japan than in Western countries (eg, [20-22]). To our knowledge, this assumption has not been evaluated empirically. Some studies have contrasted the relative frequency of stigmatising attitudes in Japan and other countries [12,14]. However, this research has not involved Western countries and suffers from methodological limitations, such as small, selected samples, as noted above. In addition, the countries studied differed in standard of living, which may affect outcome [23] and therefore indirectly, the prevalence of stigma. Moreover, it is not known whether the patterns of stigma in Japan differ from Western countries as a function of specific types of stigmatising attitude or type, severity or chronicity of mental disorder.

In this study we compare public attitudes to mental illness in Japan and Australia for depression, depression with suicidal ideation, early schizophrenia and chronic schizophrenia. This paper represents the third in a series reporting the results of a survey of mental health and related attitudes in Japan and Australia. The first paper focused on recognition of mental disorders and beliefs about the helpfulness of treatments [24] and the second on perceived risk factors and causes of depression [25]. The current paper is concerned with stigma. In particular, we test the hypotheses that stigma is more prevalent in Japanese society compared to the Australian community, that the pattern of the stigma will differ in the two countries, that stigma will be more pronounced for schizophrenia compared to depression and that stigma will be greater in the case of severe or chronic conditions compared to mild or early stage illness.

## Methods

### Survey interview

The interview questionnaire, which was developed for Australia and Japan, comprised a common core of questions and some items that were country-specific [24,25].

Each participant was presented, on a random basis, with either a female ('Mary') or male ('John') version of one of four vignettes of a person with a mental disorder. The vignettes involved either (i) a major depressive disorder; (ii) a major depressive disorder and suicidal thoughts; (iii) early schizophrenia; or (iv) chronic schizophrenia (see Appendix). Each of the disorders depicted in the vignettes satisfied the diagnostic DSM-IV and ICD-10 criteria for either major depressive disorder or schizophrenia. The vignette involving depressive disorder without suicidal thoughts and early stage schizophrenia satisfied these criteria at a minimal level in order to assess public attitudes to early-stage mental disorder. The two vignettes involving major depressive disorder were identical except one involved a person with suicidal thoughts. This enabled an assessment of the impact of suicidal thoughts on public attitudes. The two vignettes involving schizophrenia were incorporated to evaluate if public attitudes were more negative about a severe, chronic disorder compared to a less severe disorder in its early stages.

Respondents were asked a number of questions about the vignette to determine if they recognised the mental disorder it depicted, and to document their beliefs about risk factors, treatments and the prognosis for the condition, their level of contact with people such as the person in the vignette, their awareness of depictions of mental illness in the media, and their level of stigma and perceived stigma towards the person in the vignette. The survey also included questions to determine the health and socio-demographic characteristics of the respondent. The current paper focuses on stigmatising attitudes and social distance and in particular the respondents' willingness to socialise and work with the person in the vignette, and their attitudes and their perception of societal attitudes to the person in the vignette.

#### Personal and perceived stigma

Stigma was measured using two 9-item scales [26]. The first scale assessed the respondent's personal attitudes towards the person described in the vignette (Personal stigma). The second scale assessed the respondent's beliefs about other people's attitudes towards the person described in the vignette (Perceived stigma). Both scales contained essentially the same statements, but differed in terms of whether they were aimed at personal attitudes or the perceived attitudes of others. An example of a statement from the Personal stigma scale was: 'John's problem is not a real medical illness'. The corresponding statement in the Perceived stigma scale is '*Most *people believe that John's problem is not a real medical illness'. Ratings for each item of each test were made on a 5-point Likert scale (1 = strongly agree, 2 = agree, 3 = neither agree nor disagree, 4 = disagree, 5 = strongly disagree). In this paper, for each item on each scale, the categories "agree" and "strongly agree" were combined and analysed.

#### Perceived discrimination

Perception of discrimination was examined using the question 'Do you think that John/Mary would be discriminated against by others in the community, if they knew about the problems he/she has had?' The possible responses to this question were 'yes', 'no' and 'I don't know'.

#### Attitudinal social distance

Self-reported willingness to make contact with the person in the vignette was measured using a 5-item scale developed by Link, Phelan, Bresnahan, et al, 1999 [27]. In particular, respondents rated their willingness to 1) move next door to the person in the vignette; 2) spend an evening socialising with the person; 3) make friends with the person; 4) work closely on a job with the person; and 5) have the person marry into the family. Following Link et al, the Australian version of the test required the respondent to rate each item on a 4-point scale: definitely willing, probably willing, probably unwilling and definitely unwilling. The Japanese version of the scale incorporated an additional stronger rating option for which the literal translation was 'definitely not want to do that'. This category was combined with the 'definitely unwilling' category for the purposes of the current analysis.

### The Australian survey

A household survey of 3998 Australian adults aged 18 years or over was conducted by ACNielson over the period late 2003 to early 2004. Sampling covered 250 census districts, all states and territories and both rural and metropolitan areas. Interviewers made up to 5 call backs to metropolitan areas and 3 callbacks to rural areas. The target interviewee was the householder with the most recent birthday. In order to achieve the intended target of 4000 participants, visits were made to 28, 947 households. The following outcomes were achieved: no contact (n = 14,630 householders), vacant lot (306), refused (7815), temporarily unavailable target participant (1132), no suitable respondent in the household (287), did not speak English (383), incapable of responding (213), and unavailable for duration of the survey (181). Of the 3998 participants, 1001, 999, 997, and 1001 received the depression, depression with suicidal ideation, early schizophrenia and chronic schizophrenia vignettes respectively.

Ethics approval for the study was granted by the Human Research Ethics Committee of the Australian National University. Further details of the survey can be found in two previous publications [24,25].

### The Japanese survey

The Japanese survey comprised home visit interviews of 2000 adults aged 20 to 60 years and was conducted by the Yamate Information Processing Centre during the period 19 November to 12 December 2003. A survey manual supplied from Australia was translated into Japanese for use with the target population and the Japanese survey employed as far as practicable the same procedures as Australia. In order to verify the accuracy of the Japanese translation of the survey, questions developed by the Australian researchers (AFJ, HC, KMG) were translated into Japanese and then retranslated into English by a native English translator who had not sighted the original version in order to verify the accuracy of the Japanese translation. In addition to the questions taken from the Australian survey, the Japanese survey included questions concerning such issues as psychiatric health and welfare policy, the bodies implementing related services, the existence of action groups, and the change in the Japanese name for schizophrenia by the Japanese Society of Psychiatry and Neurology from 'seishinbunretusho byo' (split mind disorder) to 'tougo shittchou shou' (integration disorder).

Five hundred people received each of the four vignettes (250 for each gender-vignette combination). The survey was conducted in 25 regional survey sites throughout the country (80 households per site). It was not feasible to conduct a nationally representative sample due to time, personnel and financial constraints. It was therefore decided to sample a range of areas which varied in terms city size (large or small), prevalence of clinical or psychiatric cases (many or few) and prevalence of suicide rate (high or low). Additional reasons for selection of regional sites were that they were places of comparatively high population within the relevant regions, that survey interviewers could use public transport, and that urban areas involved were convenient for the researchers to visit within a certain range using public transportation. Since the survey was conducted during winter, and because it was difficult to ensure that there would be sufficient interviewers, the implementation of the survey in Hokkaido and Shikoku prefectures proved troublesome. Briefing meetings were held with interviewers in each region prior to commencement of the survey. Remuneration of 1000 yen was provided for each participant. Data were not collected on the refusal rate for the survey.

### Statistical analysis

Data were pooled across male and female versions of each vignette and percent frequencies calculated. For the Australian survey, percentages were calculated applying survey weights to provide more accurate population estimates. 95% confidence intervals (CIs) for these percentages were also estimated using the Complex Samples procedure in SPSS 12.0. This procedure takes account of sampling weights and geographic clustering in the sample. Percentages and 95% confidence intervals for the Japanese sample were calculated using unweighted data with SPSS 12.0. Differences between stigma in the two countries or between stigma for vignette pairs might be considered statistically significant if there is no overlap between the 95% confidence intervals for the relevant percentages. Theoretical and computer simulation studies show that this is equivalent to a pairwise comparison error rate of approximately 1% [28]. Even using this conservative approach to significance testing, it is likely that a small number of comparisons will be spuriously significant due to the large numbers of comparisons involved. There is also the likelihood that very small, and not necessarily meaningful, effect sizes will be significant due to the large sample size employed and the contribution of language differences between the two countries. Accordingly, only those comparisons which attain at least a small effect size (Cohen's h ≥0.2 [29]) are discussed in this paper. In addition the paper focuses on patterns of findings rather than isolated effects.

## Results

### Personal stigma

#### Comparison between levels of stigma in Japan and Australia

Table 1 presents the results from the personal stigma scale for the Australian and Japanese surveys. In general, stigmatising attitudes were less common among the Australian than the Japanese respondents. Compared to Australian respondents, Japanese interviewees more frequently indicated that the person in the vignette: could snap out of their problem; was suffering from a personal weakness; and did not have a real medical illness. Japanese participants were also more likely to say that they would not employ or vote for a person with such a problem. On the other hand, Australian respondents were more likely than Japanese respondents to agree that people with a mental disorder were unpredictable. Interestingly, although Japanese respondents were more likely than Australian respondents to perceive people with chronic schizophrenia as dangerous and to be avoided, there was no difference in the relative frequency with which interviewees in the two countries said that people with depression were dangerous or to be avoided.

**Table 1:** Percentage of Australian and Japanese respondents who 'agree' or 'strongly agree' with each statement from the Personal stigma scale^†^

**Statement**	**Depression Vignette**	**Depression/Suicidal Vignette**	**Early Schizophrenia Vignette**	**Chronic Schizophrenia Vignette**
**Person could snap out of the problem**				
Australian	24.7 (21.7%–27.9%)	22.7 (19.9%–25.7%)	18.3 (15.6%–21.3%)	17.9 (15.0%–21.2%)
Japanese	47.2 ^* d ^(42.8%–51.6%)	49.4 ^* d ^(45.0%–53.8%)	41.2* (36.9%–45.5%)	36.4 ^* a,b ^(32.2%–40.6%)
**Problem is a sign of personal weakness**				
Australian	13.4 (11.0%–16.2%)	16.9 (14.4%–19.6%)	19.3 (16.6%–22.4%)	14.0 (11.7%–16.7%)
Japanese	45.4* (41.0%–49.8%)	45.0* (40.6%–49.4%)	46.6* (42.2%–51.0%)	46.0* (41.6%–50.4%)
**Problem is not a real medical illness**				
Australian	14.6 (12.3%–17.1%)	15.2 (13.0%–17.8%)	14.9 (12.7%–17.4%)	13.9 (11.7%–16.6%)
Japanese	40.2* (35.9%–44.5%)	38.4* (34.1%–42.7%)	31.4* (27.3%–35.5%)	35.8* (31.6%–40.0%)
**People with this problem are dangerous**				
Australian	11.9 ^c,d ^(9.8%–14.3%)	18.3 ^c ^(15.7%–21.2%)	24.9 ^a,b ^(22.2%–27.8%)	22.5 ^a ^(19.6%–25.7%)
Japanese	14.6 ^d ^(11.5%–17.7%)	16.0 ^d ^(12.8%–19.2%)	20.4 ^d ^(16.9%–23.9%)	37.6 ^* a,b,c ^(33.3%–41.9%)
**Avoid people with this problem**				
Australian	6.9 (5.3%–9.0%)	4.7 (3.5%–6.4%)	4.9 (3.6%–6.6%)	5.2 (3.9%–7.0%)
Japanese	7.8 ^d ^(5.4%–10.2%)	5.8 ^c,d ^(3.7%–7.9%)	11.8 ^*,b ^(9.0%–14.6%)	17.8 ^* a,b ^(14.4%–21.2%)
**People with this problem are unpredictable**				
Australian	42.2 ^c,d ^(38.8%–45.6%)	51.1 ^c,d ^(47.6%–54.5%)	67.1 ^a,b ^(63.9%–70.1%)	67.5 ^a,b ^(64.1%–70.7%)
Japanese	18.6 ^* c,d ^(15.2%–22.0%)	20.0 ^* c,d ^(16.5%–23.5%)	31.0 ^* a,b,d ^(26.9%–35.1%)	45.6 ^* a,b,c ^(41.2%–50.0%)
**If I had this problem I wouldn't tell anyone**				
Australian	17.0 ^c,d ^(14.5%–19.9%)	21.5 ^d ^(18.8%–24.3%)	26.7 ^a ^(23.8%–29.8%)	31.1 ^a,b ^(28.1%–34.3%)
Japanese	26.8 ^* d ^(22.9%–30.7%)	24.8 ^c,d ^(21.0%–28.6%)	35.0 ^b ^(30.8%–39.2%)	37.2 ^a,b ^(32.9%–41.5%)
**I would not employ someone with this problem**				
Australian	21.6 ^d ^(19.2%–24.3%)	22.5 ^d ^(19.8%–25.4%)	24.8 (22.0%–27.9%)	32.4 ^a,b ^(29.2%–35.8%)
Japanese	38.6 ^* d ^(34.3%–42.9%)	38.6 ^* d ^(34.3%–42.9%)	47.6 ^* d ^(43.2%–52.0%)	61.2 ^* a,b,c ^(56.9%–65.5%)
**I would not vote for a politician with this problem**				
Australian	30.1 ^d ^(26.9%–33.4%)	32.5 ^d ^(29.4%–35.8%)	35.3 ^d ^(32.2%–38.5%)	45.7 ^a,b,c ^(42.5%–49.0%)
Japanese	58.0 ^* d ^(53.7%–62.3%)	53.8 ^* d ^(49.4%–58.2%)	58.0 ^* d ^(53.7%–62.3%)	73.8 ^* a,b,c ^(69.9%–77.7%)

^†^symbols flagging table entries denote a small (Cohen's h ≥ 0.2) to moderate (Cohen's h ≥ 0.5) effect size relative to ^a ^depression; ^b^depression with suicidal ideation; ^c^early schizophrenia; ^d^chronic schizophrenia; *Australian prevalence

Unbolded symbols indicate a small effect size. Bolded symbols denote a moderate effect size.

#### Pattern of endorsement for stigma items

The rank order of most to least endorsement across the personal stigma items was broadly similar for the four vignettes. Within each vignette, avoiding the person was the item least endorsed by Australian respondents (endorsement ranging from 4.7% for depression/suicidal vignette to 6.9% for the depression vignette). The item most commonly endorsed by Australians was that "People with the problem are unpredictable" (range 42.2% for depression to 67.5% for chronic schizophrenia). The next most endorsed item was "I would not vote for a politician with this problem" (30.1% for depression to 45.7% for chronic schizophrenia). Similarly, for the Japanese sample, personal stigma was least for avoidance (range 5.8% depression/suicidal vignette to 17.8% chronic schizophrenia) and most for voting for a politician (53.8% depression/suicidal vignette to 73.8% chronic schizophrenia). Overall, Japanese respondents more frequently endorsed personal weakness than unpredictability as attributes of the people in the vignettes, whereas the reverse was the case for the Australian respondents.

#### Comparison between vignettes

##### Depression vignettes

Australian respondents showed very similar attitudes with respect to the two depression vignettes, as did the Japanese respondents.

##### Schizophrenia vignettes

Interviewees in the Australian survey also showed similar attitudes with respect to the two schizophrenia vignettes, although an Australian respondent was less likely to vote for a person with the chronic condition. The Japanese respondents showed greater levels of personal stigma with respect to chronic than early schizophrenia on several items. Fewer were prepared to employ or vote for a person with chronic than early schizophrenia and more perceived the person with chronic compared to early schizophrenia as dangerous and unpredictable.

##### Depression vs schizophrenia vignettes

Australian interviewees tended more often to endorse stigmatising statements about one or both of the schizophrenia vignettes compared to one or both of the depression vignettes. However, there was no or very little difference in how commonly Australian participants believed that schizophrenia compared with depression was not a real medical illness, or was a sign of personal weakness, that the person with the problem was to be avoided or that the person could snap out it. The pattern of difference between the depression and schizophrenia vignettes for the Japanese respondents was broadly similar to that for the Australian respondents. However, Japanese respondents more often endorsed avoidance as a response to the person with schizophrenia than to the person with depression.

### Perceived stigma

Tables 2 show the results for the perceived stigma set of questions for the Australian and Japanese surveys. Overall, perceived stigma was substantial, with percentage of respondents endorsing a statement from the Perceived stigma scale ranging from 35.6% to 85.2% among the Australian respondents and 30% to 82% for the Japanese respondents.

**Table 2:** Percentage of Australian and Japanese respondents who 'agree' or 'strongly agree' with each statement from the Perceived stigma scale^†^

**Social Situation**	**Depression Vignette**	**Depression/Suicidal Vignette**	**Early Schizophrenia Vignette**	**Chronic Schizophrenia Vignette**
**Person could snap out of the problem**				
Australian	58.9 ^d ^(55.4%–62.4%)	59.9 ^d ^(56.5%–63.2%)	51.6 (48.5%–54.8%)	47.9 ^a,b ^(44.4%–51.4%)
Japanese	45.4 ^* d ^(41.0%–49.8%)	38.2* (33.9%–42.5%)	37.0* (32.8%–41.2%)	35.4 ^* a ^(31.2%–39.6%)
**Problem is a sign of personal weakness**				
Australian	52.6 (49.2–56.0%)	55.2 (51.9%–58.5%)	52.0 (48.7%–55.3%)	51.4 (47.9%–54.9%)
Japanese	58.2 (53.9%–62.5%)	54.2 (49.8%–58.6%)	57.4 (53.1%–61.7%)	57.8 (53.5%–62.1%)
**Problem is not a real medical illness**				
Australian	52.4 (49.1–55.7%)	54.2 (50.9%–57.4%)	47.1 (43.9%–50.4%)	47.4 (44.0%–50.8%)
Japanese	45.0 (40.6%–49.4%)	38.8* (34.5%–43.1%)	38.8 (34.5%–43.1%)	46.2 (41.8%–50.6%)
**People with this problem are dangerous**				
Australian	37.8 ^c,d ^(34.5%–41.3%)	42.1 ^c,d ^(38.9%–45.4%)	58.5 ^a,b ^(55.1%–61.8%)	60.2^a,b ^(56.6%–63.7%)
Japanese	32.6 ^c,d ^(28.5%–36.7%)	30.0 ^* c,d ^(26.0%–34.0%)	51.4 ^a,b,d ^(47.0%–55.8%)	63.6 ^a,b,c ^(59.4%–67.8%)
**Avoid people with this problem**				
Australian	35.6^d ^(32.3%–39.0%)	37.3 (34.1%–40.6%)	39.8 (36.5%–43.2%)	46.1^a ^(42.3%–50.0%)
Japanese	32.2 ^c,d ^(28.1%–36.3%)	30.2 ^c,d ^(26.2%–34.2%)	51.4 ^* a,b ^(47.0%–55.8%)	59.0 ^* a,b ^(54.7%–63.3%)
**People with this problem are unpredictable**				
Australian	65.6 ^c,d ^(62.3%–68.8%)	68.7 ^c,d ^(65.5%–71.8%)	78.4 ^a,b ^(75.3%–81.2%)	82.5 ^a,b ^(79.6%–85.1%)
Japanese	35.8 ^* c,d ^(31.6%–40.0%)	36.4 ^* c,d ^(32.2%–40.6%)	58.6 ^* a,b ^(54.3%–62.9%)	67.4 ^* a,b ^(63.3%–71.5%)
**If they had this problem most people wouldn't tell anyone**				
Australian	63.1 (59.8%–66.3%)	67.1 (63.8%–70.2%)	67.7 (64.3%–70.9%)	71.4 (68.0%–74.6%)
Japanese	37.8 ^* c,d ^(33.5%–42.1%)	35.2 ^* c,d ^(31.0%–39.4%)	49.8 ^* a,b ^(45.4%–54.2%)	48.4 ^* a,b ^(44.0%–52.8%)
**Most people would not employ someone with this problem**				
Australian	69.1 ^d ^(66.1%–72.0%)	72.3 ^d ^(69.1%–75.3%)	76.7 ^d ^(73.6%–79.5%)	85.2 ^a,b,c ^(82.6%–87.4%)
Japanese	65.6 ^b,d ^(61.4%–69.8%)	55.2 ^* a,c,d ^(50.8%–59.6%)	72.8 ^b ^(68.9%–76.7%)	79.2 ^a,b ^(75.6%–82.8%)
**Most people would not vote for a politician with this problem**				
Australian	69.0 ^d ^(65.9%–72.0%)	70.8 ^d ^(67.5%–73.8%)	76.6 (73.6%–79.5%)	83.7 ^a,b ^(81.0%–86.1%)
Japanese	73.6 ^b,d ^(69.7%–77.5%)	63.6 ^a,c,d ^(59.4%–67.8%)	72.0 ^b,d ^(68.1%–75.9%)	82.0 ^a,b,c ^(78.6%–85.4%)

^†^symbols flagging table entries denote a small (Cohen's h ≥ 0.2) to moderate (Cohen's h ≥ 0.5) effect size relative to ^a ^depression; ^b ^depression with suicidal ideation; ^c^early schizophrenia; ^d^chronic schizophrenia; *Australian prevalence.

Unbolded symbols indicate a small effect size. Bolded symbols denote a moderate effect size.

#### Comparison between levels of perceived stigma in Japan and Australia

In contrast to the findings for personal stigma, Australians were more likely than Japanese participants to agree that others in the community would have a stigmatising attitude to the person with a mental disorder. This was the case for 15 of the 36 item-vignette combinations. Moreover, there was no difference in perceived stigma between the respondents in the two countries for most of the remaining item-vignette combinations. The exception was that it was significantly more common for Japanese than Australian respondents to believe that other people would avoid a person with schizophrenia.

#### Pattern of endorsement for stigma items

The rank order of most to least endorsement across the 9 perceived stigma items was broadly similar for each of the vignettes for the Australian respondents. Australians most often endorsed the views that other people would not employ someone with the problem illustrated in the vignette (69.1% for depression to 85.2% for chronic schizophrenia), not vote for a politician with the problem (69% depression to 83.7% chronic schizophrenia) and would believe the person was unpredictable (65.6% depression to 82.5% chronic schizophrenia). The least endorsed items were that others would avoid people with the problem (35.6% depression/suicidal ideation to 46.1% chronic schizophrenia vignette), and for depression, that people with the problem are dangerous (11.9% depression to 18.3% depression/suicidal ideation). Similarly, Japanese participants most often endorsed the items that others would not vote for a politician (63.6% for depression/suicidal vignette to 82% chronic schizophrenia vignette), nor employ a person with the problem (55.2% for depression suicidal ideation vignette to 79.2% chronic schizophrenia vignette). There were some differences in the rank ordering of items for the different vignettes for Japanese participants. As was the case for the Australian participants, they least often indicated that others would avoid people with depression (30.2%–32.2%), and that they would believe people with depression are dangerous (30.0%–32.6%). The least endorsed items for the schizophrenia vignettes were that others would believe that the person could snap out it (37.0%–35.4%) and the problem was not a real medical illness (38.8%–46.2%).

#### Comparison between vignettes

##### Depression vignettes

The percentage of Australian respondents who believed others held stigmatising attitudes was strikingly similar for the two depression vignettes. There were no differences between the depression vignettes for any of nine perceived stigma items. Japanese respondents also showed similar responses for the two depression vignettes for most items, but were somewhat more likely to think that others would not employ or vote for the person with suicidal ideation compared with the person with depression alone.

##### Schizophrenia vignettes

The level of perceived stigma did not differ markedly for the two schizophrenia vignettes for most items for respondents from either country.

##### Depression vs schizophrenia vignettes

For a number of item-vignette combinations, Australian participants more often reported that others would hold a stigmatising view about a person with schizophrenia than a person with depression. In particular, a greater proportion of respondents believed that the person with schizophrenia would be seen by others as dangerous and unpredictable. Respondents were also more likely to endorse that others would employ and vote for a person with a depressive disorder compared to a person with chronic schizophrenia. Conversely, Australian participants more often believed that people with depression would be seen by others as capable of 'snapping out of it' compared to a person with chronic schizophrenia. The pattern of findings was broadly similar for the Japanese respondents except that Japanese respondents were also more likely to believe that people with schizophrenia would be seen by others as someone to be avoided and as having an illness that they would not tell anyone about.

### Comparison between personal and perceived stigma

More Australians believed that others would hold a stigmatising attitude (perceived stigma) than reported that they themselves endorsed a stigmatising view (personal stigma). This was the case for each of the stigma items and the effects were moderate to large for all but one item. Perceived stigma was also greater than personal stigma for most item-vignette combinations for the Japanese sample. The exceptions were for the items 'not a real medical illness', and 'snap out of their problem' where the levels of personal and perceived stigma were similar.

The tendency to show greater perceived than personal stigma was most marked among the Australian respondents. This trend was seen for 33 of the 36 item-vignette combinations. Only in 4 contrasts (25%) did the effect size for the discrepancy between personal and perceived stigma fall below a moderate effect for the Australian respondents, whereas 24 of the 36 contrasts (75%) fell below this level for the Japanese respondents.

### Perceived discrimination

The responses to the discrimination question are shown in Table 3. The percentage of Australians who agreed that the person in the vignette would be discriminated against by others in the community ranged from 53.5% (depression vignette) to 83.2% (chronic schizophrenia vignette). The comparable figures for the Japanese respondents were 27.6% (depression) to 62.6% (chronic schizophrenia). For each vignette, more Australian than Japanese respondents agreed that the person would be discriminated against by others in the community.

**Table 3:** Percentage of Australian and Japanese respondents who think the person described in the vignette would be discriminated against by others in the community^†^

**Social Situation**	**Depression Vignette**	**Depression/Suicidal Vignette**	**Early Schizophrenia Vignette**	**Chronic Schizophrenia Vignette**
Australian	53.5 ^c,d ^(49.8%–57.1%)	61.2 ^c,d ^(57.8%–64.5%)	75.9 ^a,b ^(72.5%–79.0%)	83.2 ^a,b ^(80.4%–85.7%)
Japanese	27.6^* c,d ^(23.7%–31.5%)	32.6^* c,d ^(28.5%–36.7%)	44.8 ^* a,b,d ^(40.4%–49.2%)	62.6% ^* a,b,c ^(58.3%–66.9%)

^†^symbols flagging table entries denote a small (Cohen's h ≥ 0.2) to moderate (Cohen's h ≥ 0.5) effect size relative to ^a ^depression; ^b^depression with suicidal ideation; ^c^early schizophrenia; ^d^chronic schizophrenia. *Australian prevalence

Unbolded symbols indicate a small effect size. Bolded symbols denote a moderate effect size.

Australian participants more commonly believed that discrimination would occur in the case of the person with early or chronic schizophrenia compared to the person with suicidal depression or depression. A similar pattern of findings was noted for the Japanese participants.

### Attitudinal social distance

#### Comparison between levels of social distance in Japan and Australia

Table 4 presents the results from the Social distance scale for the Australian and Japanese surveys, showing the percentage of Australians who were either 'probably unwilling' or 'definitely unwilling' to make social contact for each item and vignette. For all vignettes, a larger percentage of Japanese than Australian respondents were unwilling to live next door to, socialise or make friends with, work closely with or marry into the family of someone with a mental disorder. The average effect size calculated across all vignettes and items was large (1.1).

**Table 4:** Percentage of Australian and Japanese respondents unwilling to socially interact with each person described in the vignette^†^

**Social Interaction**	**Depression Vignette**	**Depression/Suicidal Vignette**	**Early Schizophrenia Vignette**	**Chronic Schizophrenia Vignette**
**Live next door**				
Australian	11.7^d ^(9.6%–14.2%)	11.1^d ^(9.2%–13.3%)	15.1^d ^(12.9%–17.7%)	25.2 ^a,b,c ^(22.5%–28.1%)
Japanese	82.0^* d ^(78.6%–85.4%)	77.6^* d ^(73.9%–81.3%)	82.6* (79.3%–85.9%)	89.2 ^* a,b ^(86.5%–91.9%)
**Evening socialising**				
Australian	10.9^d ^(8.9%–13.3%)	12.2 ^d ^(10.3%–14.5%)	15.1 ^d ^(12.9%–17.8%)	26.1 ^a,b,c ^(23.0%–29.4%)
Japanese	63.0 ^* d ^(58.8%–67.2%)	58.6 ^* d ^(54.3%–62.9%)	68.6 ^* d ^(64.5%–72.7%)	81.2 ^* a,b,c ^(77.8%–84.6%)
**Make friends**				
Australian	8.0 ^d ^(6.5%–9.9%)	9.3 ^d ^(7.6%–11.4%)	12.0 ^d ^(9.9%–14.4%)	19.7 ^a,b,c ^(17.0%–22.8%)
Japanese	57.4^* d ^(53.1%–61.7%)	56.0^* d ^(51.6%–60.4%)	63.8^* d ^(59.6%–68.0%)	73.8^* a,b,c ^(69.9%–77.7%)
**Work closely**				
Australian	21.0^d ^(18.4%–23.8%)	20.0^d ^(17.3%–23.%)	23.7^d ^(20.8%–26.7%)	33.6^a,b,c ^(30.3–37.1)
Japanese	52.6^* d ^(48.2%–57.0%)	54.0 ^* d ^(49.6%–58.4%)	59.2* (54.9%–63.5%)	65.2 ^* a,b ^(61.0%–69.4%)
**Marry into family**				
Australian	28.8 ^c,d ^(25.8%–32.0%)	33.9 ^d ^(30.8%–37.2%)	39.3 ^a,d ^(36.1%–42.7%)	53.0 ^a,b,c ^(49.5%–56.6%)
Japanese	84.0 ^* d ^(80.8%–87.2%)	84.0 ^* d ^(80.8%–87.2%)	89.0* (86.2%–91.8%)	93.0 ^* a,b ^(90.8%–95.2%)

^†^symbols flagging table entries denote a small (Cohen's h ≥ 0.2) to moderate or larger (Cohen's h ≥ 0.5) effect size relative to ^a ^depression; ^b^depression with suicidal ideation; ^c^early schizophrenia; ^d^chronic schizophrenia;*Australian prevalence. Unbolded symbols indicate a small effect size. Bolded symbols denote a moderate or larger effect size.

#### Pattern of endorsement for items

For every disorder, the social interactions in which Australians were least willing to engage were marrying into the family followed by working closely with a person with a mental disorder. Among the Australian sample, for each vignette, social distance was lowest for 'making friends'. As for the Australian respondents, social distance for Japanese respondents was greatest for marrying into the family for all conditions. Similarly, in common with Australian interviewees, social distance for Japanese respondents was low relative to other items for making friends with the person with the illness. However, for all vignettes, and in direct contrast to the pattern among Australians, Japanese respondents showed the lowest social distance for working closely with a person with a mental disorder.

#### Comparison between vignettes

Social distance scores did not differ for the depression with and without suicidal ideation conditions for either the Australian or Japanese participants. However, there was consistently higher social distance with respect to the chronic compared to the early schizophrenia vignette for the Australian participants. Social distance was also more common for the chronic schizophrenia vignette than for either depression vignette for both the Australian and Japanese participants.

## Discussion

There was a consistent and marked disparity in personally held stigmatising attitudes and social distance in the two countries. In most cases, these negative attitudes were greater among the Japanese than the Australian public.

To our knowledge, this is the first study to provide data that supports the view that stigmatising attitudes towards mental disorders are more common among the Japanese public than among the public of a Western country. There are several possible explanations for the finding. The difference in stigma might be mediated, at least in part, by the differential value placed on conformity and individualism in the two countries. Since people who are mentally ill deviate from the norm it might be expected that this would impact more negatively in Japan where conformity is said to be more highly valued [30]. A second possible mechanism for the difference between the stigma in the two countries may lie in their different mix of service delivery systems. In Japan, service delivery for people with mental disorders focuses primarily on long term hospitalisation [31], whereas the Australian system emphasises de-institutionalisation and the provision of community and rehabilitation services. In a cross cultural study of public attitudes in Bali and Japan [14], it was argued that the relative unavailability of psychiatric beds in Bali may have resulted in an increase in contact between the public and people with schizophrenia, which in turn may have produced an improvement in attitudes, since contact with a person with a mental disorder is known to decrease stigma [32]. A similar argument has also been proposed by the authors of a study contrasting perceived public stigma associated with schizophrenia among teachers in Japan and Taiwan [12]. However, as the latter acknowledge, the model of institutionalisation in Japan might reflect, rather than determine community attitudes. A third possible contribution to the difference in public stigma in Australia and Japan might lie in the wider availability of public health education and stigma reduction programs in Australia over the past 10 years [33,34]. Again, however, it is possible that the presence of stigma reduction programs are in themselves an indication of lower levels of structural and community stigma.

There was a consistent finding in each country that perceived stigma was higher than personal stigma across the stigma items. People in both countries were more likely to state that others held stigmatising beliefs. This may reflect a tendency among the public to overestimate stigma in the community. If this is true, it would imply that there would be value in conducting public awareness programs that promote knowledge of the true rates of stigma in the community. The latter may encourage help seeking among people with a mental illness. However, it is also possible that the difference between personal and perceived stigma rates reflects, at least in part, a social desirability bias in which respondents are reluctant to report their true attitudes towards the person with a mental disorder [35]. Such bias might be expected to operate both in Australia and in Japan. In Japan, social behaviour is strongly determined by 'tatemae' (what the person says to maintain harmony) rather than 'honne' (what the person is really thinking).

Interestingly, perceived stigma and perceived discrimination were greater for the Australian public than the Japanese public. That is, Australians were more likely than Japanese participants to state that others in the community would have a stigmatising attitude to the person with depression or schizophrenia. They were also more likely to report that a person with depression or schizophrenia would be discriminated against by others in the community. This contrasts with the levels of personal stigma and social distance which we found were higher in Japan than Australia. How might this set of findings be explained? Again, it is possible that the perceived stigma questions were less subject to social desirability bias than personal stigma items and therefore better reflected the real levels of stigma in each community. If this were the case it might be concluded that stigma is indeed greater in Australia than in Japan. However, given the strong influence of the 'honne'/'tatemae' culture in Japan, it would be surprising if social desirability bias were higher in Australia. Of course, what is regarded as socially desirable might differ in the two cultures. Perhaps it is more socially acceptable to hold stigmatising attitudes towards people with a mental disorder in Japan. However, such a difference would not explain the differential findings for personal and perceived stigma across the two countries. Nor is it consistent with the interpretation that 'true' levels of stigma are greater in Australia than in Japan. An alternative explanation for the results is that the relatively higher level of media exposure and awareness campaigns, including 'stigma busting campaigns', in Australia has sensitised the public to the problem of the stigma of mental illness and created an exaggerated public perception of the level of stigma in the Australian community. Elsewhere we have reported that exposure to *beyondblue*, Australia's national depression initiative, was associated with an increase in the belief that discrimination would occur [36]. This supports the interpretation that anti-stigma campaigns may raise awareness of discrimination as an issue among the public.

As far as we are aware no previous studies have investigated whether suicidal ideation or suicidal behaviour impacts on stigma or whether chronicity of a mental disorder affects public attitudes to people with schizophrenia. In this study, we found that for most items, there was no or relatively little difference, between public attitudes to depression and depression with suicidal ideation. This raises the possibility that anti-stigma programs that work for depression might also generalise to the case of people with depression accompanied by suicidal ideation. On the other hand, items that might have discriminated better between the conditions (eg, "People with this problem are only seeking attention") were not incorporated into the stigma measures. Moreover, since the vignette employed in this study was confined to suicidal ideation, the results may not generalise to stigma with respect to actively suicidal behaviour.

Social distance was greater for chronic compared to early schizophrenia for the Australian public, although this effect was not strongly evident in the Personal and Perceived stigma scales. The public in both countries also showed evidence of greater social distance, greater personal stigma and greater perceived stigma for schizophrenia (particularly in its chronic form) compared to the depression vignettes. The latter results are broadly consistent with the conclusions of previous studies, including a 1996 national survey in Australia, which reported that negative attitudes towards mental disorder are more common with respect to schizophrenia than depression [37-39].

For both the Japanese and Australian public, social distance was greatest for marrying into the family for all types of mental disorder. This is consistent with the results of previous studies and the theory that willingness to interact with a person with a mental disorder decreases with level of intimacy [35,40]. The Japanese public's preference for working closely with a person with a mental disorder compared to engaging in most other interactions (a pattern that differed from that observed for the Australian public) is of interest. It is possible that the finding reflects a different hierarchy of intimacy of interactions in Japan compared to Australia. The pattern is similar to that observed in a previous study of attitudes among members of public residing in a particular health region in Japan [41]. However, the same pattern has also been reported in a representative sample of the Swiss public [42]. Thus, it cannot be assumed that any differences between Australia and Japan in the comparative prominence of social distance at work (relative to other activities) is attributable to differences inherent in a Western compared to a non-Western culture.

In each country, some stigmatising beliefs were more strongly endorsed than others. For example, in both Australia and Japan, personal stigma was least common with respect to avoiding the person with a mental disorder but among the most common for not voting for a politician with a mental disorder. (A broadly similar pattern was noted for perceived stigma except for the avoidance item with the schizophrenia vignettes for the Japanese public). At first sight, the low level of avoidance is encouraging. However, when asked about a concrete situation, such as willingness to spend an evening or working closely with a person, both the Japanese and the Australian public are less willing to interact with people with a mental disorder. This discrepancy may reflect a social desirability bias in the responses to the 'avoidance' question. It is equally possible that the concept of 'avoidance' is conceptualised by the public at a different, much less intimate level of social interaction (eg, not crossing to the other side of the street or walking out a shop on the appearance of a person with a mental disorder) than that specified in the social distance scale. Alternatively, concrete examples may elicit responses that are more closely aligned with behaviour than a statement seeking a general attitude.

This study identified high levels of personal and perceived stigma in the domain of employment recruitment for both countries. It may be that employers are a possible target group for awareness and anti-stigma campaigns, although care would need to be taken that such campaigns did not increase discriminatory behaviour through improved employer recognition and therefore exclusion of people with mental disorders in the workplace [27].

Finally, although there are similarities between the Japanese and Australian public in the pattern of item endorsement, there were also differences. The Australian public strongly endorsed unpredictability as a characteristic of people with depression and schizophrenia whereas 'personal weakness' was among the most endorsed characteristic by the Japanese public. We have previously reported that 'weakness of character' was more often endorsed as a risk factor for mental disorder by the Japanese than the Australian respondents [25]. These findings may point to ways in which interventions programs for reducing stigma might be tailored for each country, with particular emphasis for example placed on unpredictability in the Australian context and more emphasis placed on combating the attitude that mental illness reflects a 'personal weakness' in Japan.

### Limitations

This study has a number of limitations. First, the rate of non-contact in the Australian survey is of some concern. Secondly, the Japanese survey was not a representative national survey. Thirdly, responses may have been affected by social desirability biases. Fourth, the personal and perceived stigma items were devised for use in evaluating depression and may not be optimal for detecting patterns of stigma in other disorders. Fifth, the rating scales for the social distance item differed. Finally, cross-cultural comparisons may reflect differences in interpretation of questions, and cultural factors that affect interviewee responses.

## Conclusion

In this study we have documented both similarities and differences in public attitudes to mental illness in Japan and Australia. The marked discrepancy between personal and perceived stigma among members of the public in both countries, suggests that there may some value in disseminating normative information about personal stigma to the public in an effort to promote help seeking in both countries. In addition, given the high level of personal stigma among the Japanese public, there may be value in developing and evaluating public interventions designed to reduce the stigma of mental illness in Japan. Clearly, such interventions must be culturally appropriate and may differ from programs developed in Western countries which emphasise individualist approaches [43]. Nevertheless, there is some evidence to suggest that educational programs can improve negative attitudes to mental illness among the Japanese public [44]. Finally, the study reported here represents the first systematic national survey of stigma and attitudinal discrimination in Australia and Japan. The current work provides a baseline for tracking the level of stigma on a national basis into the future. The information from such surveys could provide invaluable information to policy makers in both countries.

## Competing interests

The author(s) declare that they have no competing interests.

## Authors' contributions

KMG: Wrote the paper and co-designed the Australian survey

YN: Provided overall supervision of the research and comments on the manuscript

HC: Co-designed the Australian survey and edited the paper

KY: Provided specific guidance on the Japanese survey and co-wrote the methods section of the manuscript

AFJ: Co-designed the Australian survey, co-wrote the method section and edited the paper

HN: Was involved in the coordination between the Japanese and Australian surveys and co-wrote the method section of the manuscript

## Appendix. The vignettes

### (a) Depression vignette (John version)

John is 30 years old. He has been feeling unusually sad and miserable for the last few weeks. Even though he is tired all the time, he has trouble sleeping nearly every night. John doesn't feel like eating and has lost weight. He can't keep his mind on his work and puts off making decisions. Even day-to-day tasks seem too much for him. This has come to the attention of his boss, who is concerned about John's lowered productivity.

### (b) Depression vignette with suicidal thoughts (John)

John is 30 years old. He has been feeling unusually sad and miserable for the last few weeks. Even though he is tired all the time, he has trouble sleeping nearly every night. John doesn't feel like eating and has lost weight. He can't keep his mind on his work and puts off making any decisions. Even day-to-day tasks seem too much for him. This has come to the attention of john's boss who is concerned about his lowered productivity. John feels he will never be happy again and believes his family would be better off without him. John has been so desperate, he has been thinking of ways to end his life.

### (c) Early schizophrenia vignette (John)

John is 24 and lives at home with his parents. He has had a few temporary jobs since finishing school but is now unemployed. Over the last six months he has stopped seeing his friends and has begun locking himself in his bedroom and refusing to eat with the family or to have a bath. His parents also hear him walking about his bedroom at night while they are in bed. Even though they know he is alone, they have heard him shouting and arguing as if someone else is there. When they try to encourage him to do more things, he whispers that he won't leave home because he is being spied upon by the neighbour. They realize he is not taking drugs because he never sees anyone or goes anywhere.

### (iv) Chronic schizophrenia vignette (John)

John is 44 years old. He is living in a boarding house in an industrial area. He has not worked for years. He wears the same clothes in all weathers and has left his hair to grow long and untidy. He is always on his own and is often seen sitting in the park talking to himself. At times he stands and moves his hands as if to communicate to someone in nearby trees. He rarely drinks alcohol. He speaks carefully using uncommon and sometimes made-up words. He is polite but avoids talking with other people. At times he accuses shopkeepers of giving information about him to other people. He has asked his landlord to put extra locks on his door and to remove the television set from his room. He says spies are trying to keep him under observation because he has secret information about international computer systems which control people through television transmitters. His landlord complains that he will not let him clean the room which is increasingly dirty and filled with glass objects. John says he is using these "to receive messages from space".

## Pre-publication history

The pre-publication history for this paper can be accessed here:


